# Addressing Scanxiety Throughout the Cancer Surveillance Journey: A Co-Design Study

**DOI:** 10.3390/healthcare14203536

**Published:** 2026-10-20

**Authors:** Hannes Vanpoecke, Kaat De Pourcq, Paul Gemmel, Bert R. Meijboom, Melissa De Regge

**Affiliations:** 1 Center for Service Intelligence, Department of Marketing, Innovation and Organisation, Faculty of Economics and Business Administration, Ghent University, Tweekerkenstraat 2, 9000 Ghent, Belgium; kaat.depourcq@ugent.be (K.D.P.); paul.gemmel@ugent.be (P.G.); melissa.deregge@uzgent.be (M.D.R.); 2 Department Tranzo, Tilburg University, 5037 AB Tilburg, The Netherlands; b.r.meijboom@tilburguniversity.edu; 3 Strategic Policy Cell, Ghent University Hospital, 9000 Ghent, Belgium

**Keywords:** scanxiety, patient journey, co-creation, journey redesign, cancer survivorship, supportive cancer care

## Abstract

**Highlights:**

**What are the main findings?**

- Scanxiety unfolds throughout the entire cancer surveillance journey, spanning three phases and seven subphases, rather than being limited to the imaging procedure alone.
- Participants generated intervention opportunities that focused on refining existing touchpoints, adding supportive resources, and eliminating unnecessary sources of burden across multiple dimensions of the cancer surveillance experience.

**What are the implications of the main findings?**

- Efforts to reduce scanxiety should extend beyond psychosocial interventions around scans and also address administrative and organisational factors such as coordination, waiting times and the delivery of results.
- Cancer surveillance services should provide flexible, patient-centred support options, co-designed with survivors and other stakeholders recognising that support preferences vary across individuals and throughout the surveillance journey.

**Abstract:**

**Background/Objectives:** Scanxiety, the distress associated with cancer surveillance imaging, is a common concern among cancer survivors. Although most research has focused on the imaging event itself, less is known about how scanxiety unfolds throughout the broader cancer surveillance journey. This study aimed to map the cancer surveillance journey of cancer survivors and identify patient-centred intervention opportunities to reduce scanxiety. **Methods:** We used a qualitative participatory design involving cancer survivors, travelling companions, and healthcare professionals. Nine co-creation workshops were conducted; in six survivors and companions participated in journey-mapping exercises, while in three, healthcare professionals completed an immersive exercise. All groups subsequently engaged in structured brainstorming workshops to generate intervention opportunities. Data were analysed using abductive thematic analysis. **Results:** Scanxiety extended beyond the imaging procedure and unfolded throughout the cancer surveillance journey, spanning three phases and seven subphases. Participants identified opportunities to redesign the journey by enhancing existing touchpoints, augmenting the journey with additional touchpoints, and eliminating unnecessary or stress-inducing touchpoints. These opportunities targeted interpersonal, technical, environmental, and administrative dimensions of care. Administrative qualities, particularly timeliness and operational processes, emerged as important contributors to distress. Preferences for support and communication varied, underscoring the need for flexible supportive care during medical surveillance. **Conclusions:** Scanxiety is a journey-wide experience shaped by both individual and organisational factors. Opportunities to address scan-related distress may include not only psychosocial support, but also improvements in how surveillance care is organised and delivered. The findings provide a patient-centred approach for redesigning cancer surveillance services and identifying intervention opportunities for future evaluation.

## 1. Introduction

Cancer survival rates have improved in recent decades, reaching up to 95.2% [1]. However, ongoing surveillance remains a significant burden for cancer survivors and their families, posing an important challenge for supportive cancer care [2]. Follow-up care often involves repeated imaging procedures and consultations to detect recurrence, monitor disease progression, or assess long-term treatment effects [3]. Although clinically necessary, these surveillance scans are frequently associated with anxiety and distress [4]. Cancer survivors (i.e., patients who have completed their primary treatment and entered the follow-up phase) may be particularly vulnerable to anxiety related to fear of recurrence or disease progression, especially given that surveillance can continue for 10 years or longer [5,6]. However, the burden of surveillance extends beyond fear of recurrence and may also include emotional distress and physical discomfort before, during, and after imaging. These experiences are collectively referred to as scanxiety [7,8,9,10]. Following a recent conceptualisation, we adopt scanxiety as a multidimensional experience shaped by cancer-related concerns as well as cognitive, behavioural, physical, and interpersonal factors [11]. Scanxiety has been linked to reduced psychosocial well-being and quality of life and is recognised as an important component of the survivorship experience [12]. Scanxiety can be triggered by fear of recurrence or progression, treatment-related concerns, and prolonged waiting times for scans and their results [4,7,13,14]. Reported prevalence rates of moderate to severe scanxiety range from 13% to 83% across cancer populations. This wide variation may be partly explained by differences in patient populations (e.g., cancer type), assessment methods, and the timing of measurement. In addition, the absence of validated scanxiety-specific measurement tools may contribute to the heterogeneity in reported prevalence rates [4,15].

Since the 2010s, considerable attention has been devoted to developing supportive care interventions for scanxiety, particularly within psycho-oncology. Existing approaches include mindfulness, relaxation, distraction techniques, massage therapy, patient education, and communication-focused interventions [16,17,18,19,20,21,22,23,24,25,26,27,28]. Although these interventions show promise, they primarily target specific moments or experiences related to surveillance imaging, rather than addressing the broader surveillance journey [29]. This is particularly relevant given that scanxiety is not confined to the imaging procedure itself. As highlighted by Khatri et al. [14] in their review of scanxiety following curative cancer surgery, distress can arise at multiple stages throughout the surveillance process. Moreover, existing interventions rarely account for how patients’ experiences and support needs may differ throughout these stages [14,29]. Building on these insights, we adopt a patient journey perspective that conceptualises the cancer surveillance journey as the follow-up trajectory of cancer survivors across recurring scans and consultations. This perspective enables scanxiety to be understood as a longitudinal experience that unfolds over time [30,31,32].

Despite growing attention to scanxiety and the development of psychosocial and educational interventions, less is known about how scan-related distress is shaped by the broader social context in which cancer surveillance occurs [7,14,33,34]. Broader organisational and system-level factors that influence the cancer surveillance journey, such as care processes, coordination, and interactions with healthcare professionals, also remain underexplored [35]. These interaction points, or touchpoints, represent the discrete moments when individuals engage with the health service system. They help structure the cancer surveillance journey, and shape individuals’ experiences, emotions, and thoughts [30]. We adopt a patient-centred perspective in which touchpoints are not limited to interactions fully under organisational control, but also include encounters within the survivors’ wider social environment, such as support from peers or companions. These non-clinical touchpoints influence survivors’ experiences of cancer surveillance, highlighting the broader social and relational context in which scanxiety occurs. Because these experiences emerge from the interaction between individual, social, and organisational factors, co-creation offers a valuable approach for identifying contextually relevant opportunities for improving the surveillance experience [35]. By actively involving survivors and other stakeholders throughout the research process, intervention opportunities can be grounded in both lived experience and care delivery realities [35,36].

This study has a twofold aim: (1) to identify the distinct phases of the cancer surveillance journey, thereby providing a clear basis for developing impactful interventions, and (2) to explore how the cancer surveillance journey can be redesigned to better align with survivors’ individual needs and preferences. This led to the following research question: What are the distinct phases of the cancer surveillance journey experienced by cancer survivors, and how can existing cancer surveillance journeys be redesigned to better align with their needs and preferences and support them in coping with scanxiety?

This study advances understanding of scan-related distress in three ways. First, it conceptualises scanxiety as a longitudinal experience that unfolds throughout the cancer surveillance journey. By identifying distinct surveillance subphases, it provides a more nuanced understanding of how distress manifests across these phases, moving beyond the conventional distinction between before, during, and after imaging. Second, through a co-design process involving multiple stakeholders, this study identifies three approaches for redesigning the cancer surveillance journey to better align with needs and preferences. Finally, it highlights the importance of administrative aspects of care, suggesting that organisational and operational features of the surveillance experience may represent underexplored opportunities for supporting individuals undergoing cancer surveillance. In doing so, we offer an understanding of scanxiety throughout the surveillance journey and contribute to the emerging supportive care literature by identifying patient-centred opportunities to improve the cancer surveillance experience.

## 2. Materials and Methods

### 2.1. Study Design

A qualitative participatory two-phase design was used between November 2024 and July 2025, comprising co-creation workshops followed by a heterogeneous focus group. The participatory approach ensured that the mapped cancer surveillance journey reflected the lived experiences of cancer survivors and supported the co-creation of contextually grounded interventions. Companions of cancer survivors were included because cancer surveillance is often experienced within a broader social context, with companions also experiencing scanxiety throughout the journey [33,37,38]. Including their perspectives enabled a broader understanding of the cancer surveillance journey and helped ensure that intervention opportunities were designed with both survivors and companions in mind. Participants contributed to the development of the surveillance journey map, the generation of intervention opportunities, and the interpretation and refinement of findings through member checking. The study adhered to the Standards for Reporting Qualitative Research (SRQR) guidelines [39].

### 2.2. Selection of Participants and Setting

Using maximum variation sampling, adult cancer survivors in follow-up for testicular cancer, breast cancer, multiple myeloma, lymphoma, or melanoma were recruited together with their companions. These cancer types were selected to ensure variation in participant demographics and surveillance trajectories, given that they are often associated with different age groups, sexes, follow-up schedules, and imaging modalities such as CT, PET-CT, MRI, and ultrasound [40]. Participant characteristics are presented in Supplementary Table S1. Inclusion criteria for survivors comprised a diagnosis of one of the specified cancers, being in follow-up (i.e., not receiving active anti-cancer treatment), and Dutch language proficiency. Companions were eligible if they were accompanying an eligible survivor and were Dutch-speaking. Recruitment proceeded via national cancer support organisations, oncology, dermatology, and radiology departments in Flemish hospitals, targeted social media, and online and physical flyers. Healthcare professionals (HCPs) were purposively recruited through professional networks and flyers to ensure a broad range of perspectives on the organisation and delivery of cancer surveillance care. Eligible participants included direct care providers in oncology (e.g., nurses, oncologists, general practitioners, and radiotherapists) and other stakeholders in oncological care (e.g., quality board members, IT nurses, oncology and psychology researchers, and pharmaceutical representatives). Workshops were hosted at four locations across Flanders, Belgium, to increase generalisability and accessibility for all participants.

### 2.3. Data Collection

The study followed a two-phase structure: (1) co-creation workshops using a journey mapping approach with two distinct groups: the first consisting of cancer survivors and their companions, and the second consisting of HCPs; and (2) a heterogeneous co-creation workshop to validate the findings from the first phase and discuss the results of a ranking questionnaire (see Table 1 for an overview of the workshops). Qualitative data were collected through sticky notes, interview transcripts, and reflective notes. These methods aimed to capture scanxiety throughout the entire cancer surveillance journey, identify key points and unmet needs from multiple stakeholder perspectives, and co-create and refine actionable intervention opportunities.

#### 2.3.1. Mapping the Cancer Surveillance Journey and Co-Creating Interventions

The lead researcher developed a preliminary version of the co-creation workshop. This version was tested with the patient representative groups as well as through two dry runs involving several researchers from different backgrounds, including marketing, health management and service research, to draw on their domain expertise and to check applicability with participants without medical training. These dry runs confirmed the suitability of the workshop for the target groups and helped identify areas where additional explanations were needed, allowing the methodology to be refined accordingly. The first round of co-creation workshops was scheduled between November 2024 and March 2025. Two distinct types of co-creation workshops were conducted with different participant groups: (1) cancer survivors and their companions and (2) HCPs (see Table 1).

The co-creation workshops began with an introduction, during which the participants and the research team introduced themselves. Participants were then divided into two groups: survivors with their companions, and HCPs. This division was made to create an open space for each group to reflect on the cancer surveillance journey from their own perspectives while minimising potential power imbalances [41]. As the study aimed to generate a comprehensive understanding of the cancer surveillance journey from diverse stakeholder perspectives, rather than comparing experiences between professional groups, HCPs were treated as a single stakeholder group despite representing different clinical, administrative, and supportive roles.

The content of these workshops was tailored to each audience. Cancer survivors and their companions were asked to share their experiences through a journey mapping exercise, in which they documented scan-related moments, emotions, and social interactions on sticky notes, thereby mapping their cancer surveillance journey. HCPs, by contrast, participated in an immersive exercise building on insights derived from these journey maps, allowing them to reflect on challenges within the cancer surveillance journey from a professional and organisational perspective. Following these group-specific activities, both workshop formats converged in a structured brainstorming exercise, following the 6-3-5 method [42]. This exercise aimed to generate potential interventions that reflected participants’ needs and preferences, and to discuss their feasibility with HCPs, with the ultimate goal of reducing scanxiety [43].

Each workshop concluded with a brief reflective discussion to summarise key insights, followed by a debriefing for the research team. Workshops were conducted with a maximum of ten participants to support active engagement [44]. With participants’ consent, all sessions were audio- and video-recorded, and artefacts from the journey mapping and 6-3-5 brainstorming exercises were photographed and securely stored.

#### 2.3.2. Phase Two: Ranking Questionnaire and In-Depth Focus Group

In May 2025, participants from the first phase were invited to complete a non-validated ranking questionnaire to support member checking and prepare for the follow-up focus group (see Supplementary Materials).

Based on the preliminary analysis of the co-creation workshops, the questionnaire included the initially identified phases and subphases of the cancer surveillance journey, as well as the grouped intervention opportunities. Participants were asked to rank the most impactful moments of the cancer surveillance journey, and the perceived relevance of the intervention opportunities, and could provide additional comments in open-ended feedback fields. In addition, the questionnaire enabled participants to provide their perspectives independently before the heterogeneous co-creation workshop, thereby reducing the risk of one perspective dominating. The questionnaire responses were used to validate the preliminary findings, identify areas requiring further discussion, and guide the agenda of the subsequent focus group. The focus group discussion was held in July 2025 with a subgroup of participants from the first phase. The lead researcher acted as a facilitator, actively encouraging participation and discussion from both groups. The purpose of the focus group was to validate and refine the emerging findings. The journey-related results were validated first, followed by a discussion of the interventions. This process largely confirmed the preliminary analysis but led to further refinement of the journey structure, resulting in the final seven-subphase cancer surveillance journey.

### 2.4. Data Analysis

Audio recordings were transcribed verbatim and analysed alongside sticky notes, reflective notes, and visual artefacts from the journey mapping and brainstorming exercises. Data analysis followed an abductive qualitative approach, beginning with a fully inductive phase and followed by a theory-informed interpretive phase [45].

In the first analytical stage, data relating to participants’ experiences, emotions, stressors, and touchpoints were analysed using reflexive thematic analysis [46]. The lead researcher initially coded the interview transcripts, visual artefacts, and sticky notes using MAXQDA 24 [47]. Through iterative comparison of these data sources, recurring patterns were identified and subsequently organised into phases and subphases of the cancer surveillance journey. Emerging interpretations were regularly discussed within the research team to allow different perspectives to inform the ongoing analysis.

In the second stage, intervention ideas generated during the co-creation workshops were analysed inductively. Through iterative comparison across data sources, intervention opportunities were initially grouped into data-driven categories reflecting shared characteristics and objectives. These categories were subsequently discussed within the research team in relation to the different phases and subphases of the cancer surveillance journey. Through this iterative process, three journey redesign approaches were identified: enhancing existing touchpoints, augmenting the journey with additional touchpoints, and eliminating unnecessary or stress-inducing touchpoints. These approaches represented higher-order interpretations that integrated the intervention opportunities with the patient journey perspective.

In the subsequent analytical stage, the analysis moved beyond the classification of journey redesign approaches. Building on the inductive coding process described above, the lead researcher identified broader quality dimensions underlying the proposed interventions. These emerging dimensions were discussed iteratively within the research team and compared with existing literature. During this theory-informed process, substantial conceptual overlap was identified with the established service quality framework of Dagger et al., which conceptualises service quality as a multidimensional and hierarchical construct reflecting patients’ overall evaluation of service, comprising four overarching dimensions: (1) interpersonal, (2) technical, (3) environmental, and (4) administrative quality [48]. As administrative aspects appeared to be central when classifying the interventions, we also identified in the data subdimensions from the service quality framework of Dagger et al. [48] such as timeliness and operations. This step connected the participants’ data-driven insights to existing theory while preserving their inductive origins and marked a shift from a purely inductive logic towards a more abductive analytical approach.

The analysis resulted in a patient-centred map of the cancer surveillance journey and two complementary lenses: redesign approaches describing how the cancer surveillance journey can be redesigned, and the service quality framework describing which experiential dimensions of care the intervention opportunities target. Interpretations were refined through iterative discussion with the senior research team and discussed with participants during member checking in the focus group.

### 2.5. Research Team and Reflexivity

The lead researcher (HV), a registered nurse and PhD candidate with experience in qualitative research, facilitated the co-creation workshops and was unfamiliar to participants prior to the study. To support reflexivity, he engaged in bracketing throughout the research process by documenting and reflecting on his preconceptions and assumptions, helping to promote transparency and awareness of potential influences on data collection and interpretation. The study was supported by a multidisciplinary research team of five researchers with expertise in healthcare operations management, clinical nursing, and service design. Workshop facilitation and data collection were further supported by four trained research assistants, who contributed reflective notes that were discussed during debriefing sessions and informed ongoing data collection and analysis.

Consistent with Braun and Clarke’s reflexive thematic analysis, data collection was not guided by the expectation that no new codes or themes would emerge. Instead, data sufficiency was judged pragmatically through ongoing assessment [49]. Regular discussions were held within the research team to evaluate whether additional data collection was continuing to provide meaningful new insights into the cancer surveillance journey or intervention opportunities. In the later co-creation workshops, contributions largely enriched and refined existing intervention categories rather than substantially broadening the conceptual scope of the analysis. The research team therefore judged the dataset to be sufficiently rich and diverse to address the study aim and ceased data collection [46].

### 2.6. Ethics

Each participant was provided with a short summary of the co-creation workshop when they signed up for the study. Written informed consent was obtained before the start of the workshop. The lead researcher reviewed the consent forms with each participant to make them aware of their rights in the study. Written informed consent was then obtained for participation and publication. Ethical approval was obtained from the Ethics Committee of Ghent University Hospital (ONZ-2024-0177).

## 3. Results

A total of 52 unique participants took part in the study: 47 joined the co-creation workshops, while an additional five eligible cancer survivors who could not attend the workshops completed the questionnaire together with 26 participants from the first round. Six of the participants from the first round of workshops (three cancer survivors and three HCPs) later took part in a focus group to discuss the preliminary results in more depth (see Supplementary Table S1).

### 3.1. Mapping the Cancer Surveillance Journey and Intervention Opportunities

**Figure 1:**
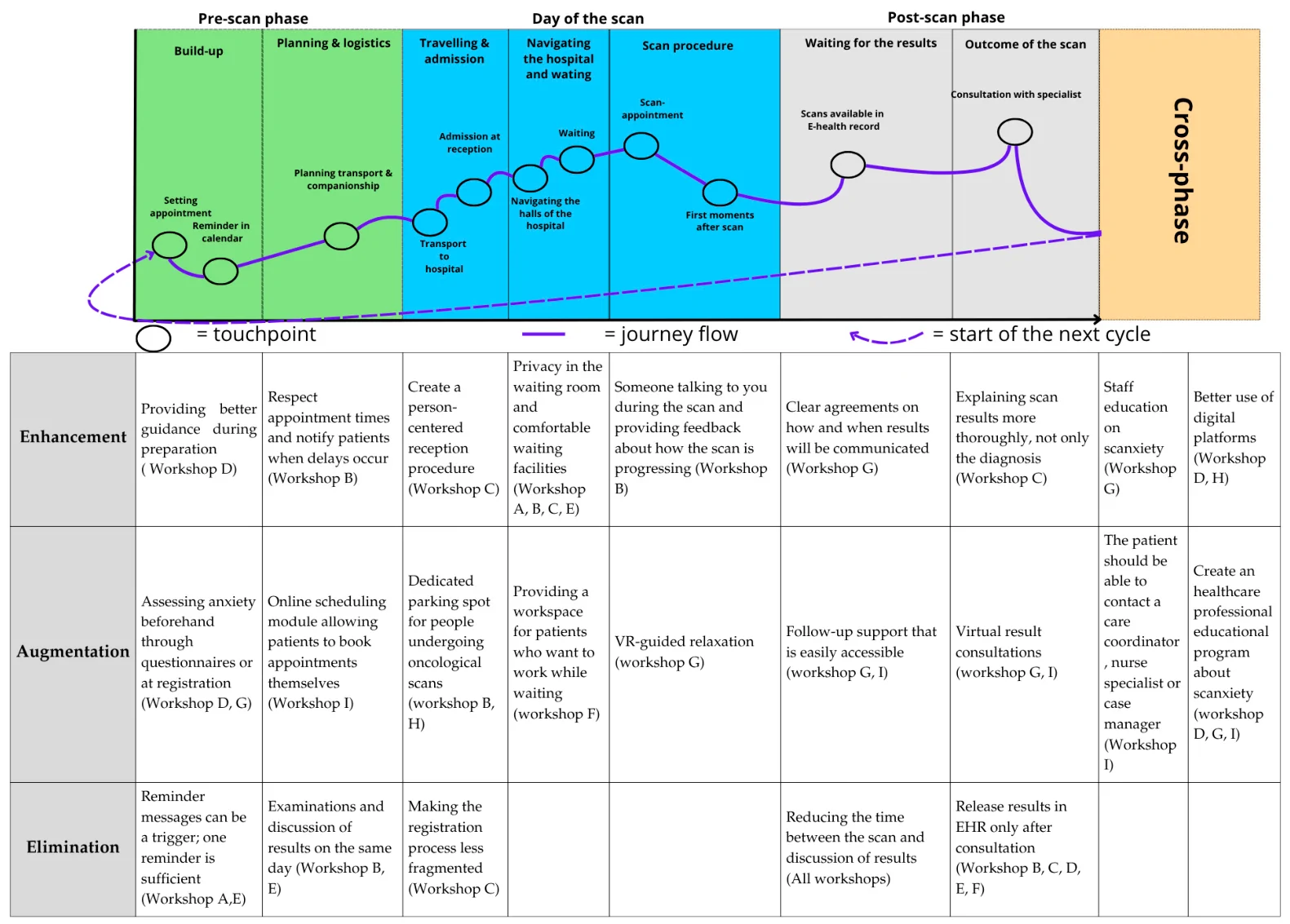
A simplified example of a cancer surveillance journey with redesign approaches and examples of intervention opportunities.

Analysis of the transcripts and visual data from the co-creation sessions revealed that scanxiety unfolds throughout the entire cancer surveillance journey rather than being confined to the scan procedure itself. Participants described seven subphases, each associated with specific stressors and emotional peaks. These subphases could be grouped into three overarching phases: pre-scan, day of the scan, and post-scan (see Figure 1). Although common patterns emerged, experiences and preferences varied between individual participants, highlighting the need for personalised approaches to surveillance care. Across all phases, participants identified opportunities to improve the surveillance experience that were directly linked to the challenges encountered throughout the journey. A more extended overview of journey phases, redesign approaches, and intervention opportunities is provided in Supplementary Table S2.

Participants generated a wide range of opportunities for improving the surveillance experience. These opportunities could be organised into three journey redesign approaches that emerged inductively from the data: **enhancement**, **augmentation**, and **elimination**. Enhancement involved improving existing touchpoints, such as communication, information provision, environments, or operational processes. Augmentation involved adding a new touchpoint, support mechanism, resource, or service that was not previously part of the surveillance journey. Elimination focused on removing touchpoints that participants perceived as unnecessary, redundant, or distress-inducing. Rather than providing fixed solutions, these approaches offer foundational principles to support the development of context-sensitive, patient-centred interventions that are tailored to the needs and experiences of individuals. These approaches are not mutually exclusive and frequently co-occur within the same phase, reflecting how scanxiety can be addressed through a combination of refining, adding to, and simplifying care experiences. Table 2 shows the redesign approaches alongside illustrative intervention opportunity examples and the redesign approach coding.

While the intervention opportunities and redesign approaches emerged inductively from the data, the health service quality dimensions proposed by Dagger et al. [48] were introduced during the subsequent theory-informed phase to organise and interpret these opportunities. Intervention opportunities targeted interpersonal, technical, environmental, and administrative care quality dimensions. The administrative dimension, particularly the subdimensions of timeliness and operations, emerged as a prominent target of intervention opportunities. Timeliness refers to temporal aspects of care such as waiting times and appointment times, whereas operations concern the coordination and organisation of care processes. Together, these dimensions illustrate how supportive care needs during cancer surveillance extend beyond psychological distress alone and are shaped by interpersonal, technical, environmental, and administrative experiences throughout the surveillance journey. This categorisation complements the journey redesign approaches by highlighting which dimensions of the care experience were addressed, while the phase-based structure of Figure 1 clarifies where these intervention opportunities are situated within the cancer surveillance journey.

### 3.2. Pre-Scan Phase

**Build-up to the scan.** Scanxiety often began when survivors became aware of the upcoming scan and intensified through repeated reminders from the hospital and cues from their social environment. Multiple reminders were frequently experienced as emotionally activating rather than reassuring. One participant remarked, *“Three or four reminders is too much. It made me think about the upcoming scan even more.”* (Survivor A). During this period, survivors actively sought information via hospital leaflets or online searches, which could provide reassurance but could also increase uncertainty and emotional distress due to missing or contradicting information.

Intervention opportunities in this subphase primarily targeted interpersonal and administrative qualities. Enhancing existing touchpoints focused on improving preparatory information, such as clearer brochures and pre-scan phone calls. Augmenting the journey involved adding informational touchpoints, including VR-preparation videos, QR-coded information, and digital tools that offered structured alternatives to unverified online searching. Eliminating touchpoints concerned reducing repeated reminders that participants associated with heightened anticipatory anxiety.

**Planning and logistics.** The planning and logistics subphase involved reorganising schedules and preparing for procedures, which introduced uncertainty and stress. Survivors raised practical concerns about medication use and fasting requirements, as illustrated by one participant who asked, *“Can I take this medication or not and from what time do I have to fast?”* (Survivor I). Travel arrangements to hospitals—often located near congested roads—and decisions about accompaniment further contributed to stress. These demands also directly affected work schedules of survivors and their social networks. Over time, repeated absences placed increasing pressure on work arrangements. As one participant noted, *“The hospital assumes my employer will accommodate this, but they can’t do that forever.”* (Survivor G).

Intervention opportunities in this subphase primarily addressed administrative qualities, particularly timeliness and operations. Enhancing existing touchpoints focused on optimising scheduling and sequencing of appointments and combining preparatory procedures to reduce fragmentation. Augmenting the journey introduced additional administrative and informational touchpoints, such as online scheduling and options outside office hours. Eliminating touchpoints focused on removing unnecessary transfers to the hospital by combining appointments to reduce logistical and administrative burden, underscoring how emotional distress in this phase was linked to operational organisation.

### 3.3. Day of the Scan

**Travelling and admission.** The journey to the hospital was often described as stressful due to traffic and parking difficulties. As one participant explained, *“Sometimes I’m driving around for half an hour to find a parking spot, and I start stressing about being late.”* (Survivor E). Admission procedures were similarly burdensome, involving multiple steps and long distances within the hospital. One participant noted, *“You have to go from central admission to admission pillars, to the waiting room where you have to scan your ticket again.”* (Survivor G).

Intervention opportunities during travelling and admission addressed a combination of administrative and environmental qualities. Enhancing existing touchpoints focused on improving operations and timeliness, such as clearer admission flows, person-centred reception procedures, and updates on delays before coming to the hospital. Augmenting the journey introduced additional support-oriented touchpoints, including parking assistance, childcare, and guides or volunteers. Eliminating touchpoints targeted administrative fragmentation by reducing multiple check-ins and unnecessary internal transfers.

**Navigating the hospital and waiting.** Several participants emphasised that unclear directions and navigating the hospital exacerbated their scanxiety, particularly when combined with time pressure. As one participant described, *“I had to walk for twenty minutes through the hospital to get to the next place. You don’t know where you’re going, and that makes you even more nervous.”* (Survivor M). Waiting rooms were described as emotionally charged environments marked by limited privacy, unpredictable delays, and a tense atmosphere. As one participant stated, *“It is such a big room… There is such a negatively charged atmosphere.”* (Survivor C).

Intervention opportunities in this subphase strongly emphasised environmental quality, particularly atmosphere and tangibles. Enhancing existing touchpoints involved clearer wayfinding, improved seating, increased privacy, and noise reduction. Interpersonal interaction was also addressed through more timely and transparent information provision. Augmenting the journey added social and emotional support elements, such as waiting-room guides, distraction spaces, and opportunities for supportive interaction. Augmentation also included interventions targeting the timeliness subdimension such as development of a digital system to communicate waiting times and delays. No intervention opportunities for eliminating touchpoints were identified in this subphase.

**Scan procedure.** Preparation for the scan involved undressing and establishing intravenous access, while companions remained in the waiting area with limited information, which heightened their distress. One companion noted, *“**You start worrying about the outcomes of your loved one. And I can’t say that I have a lot of scanxiety, because they experience the scans. But I do suffer a lot if I have to be honest”* (Companion A). The scanning environment itself was frequently described as cold and sterile. Immobility, loud equipment, and isolation contributed to discomfort: *“It is really cold. I don’t find it comforting.”* (Survivor D). For survivors, distress peaked during the scan, driven by fear of diagnostic outcomes, occasional claustrophobia, and feelings of loneliness.

Intervention opportunities during the scan mainly addressed interpersonal, environmental, and administrative qualities. Enhancing existing touchpoints focused on communication and reassurance during the exam, alongside improvements in physical comfort such as warm blankets and noise reduction. Augmenting the journey introduced survivor-controlled coping options, including music, podcasts, or VR, and expanded professional support through the availability of psychological support or age- and gender-sensitive care practices.

### 3.4. Post-Scan Phase

**Waiting for results.** Completing the scan often brought brief relief, followed by heightened anxiety due to uncertainty while waiting for the results. This period was described as the most nerve-wracking part of the journey. As one participant explained, *“Yes, the scan gives stress, but after the scan I have this feeling of relief, until reality hits again and I have to wait for the results.”* (Survivor F). Waiting times were frequently experienced as unbearable: *“We sometimes had to wait an entire week for the results, which was unbearable. It would drive you crazy.”* (Survivor K).

Intervention opportunities during this waiting period primarily targeted interpersonal and administrative qualities, particularly timeliness and operations. Enhancing existing touchpoints focused on shortening scan-to-result timelines and offering same-day discussions. Augmenting the journey added optional interpersonal and support-oriented touchpoints, such as GP-initiated contact or low-threshold psychological check-ins. Elimination involved reducing waiting-related touchpoints by shortening the period between the scan and receiving the results.

**Outcome of the scan.** Digital portals provided early access to scan images, prompting survivors to interpret results independently. This often led to distress, as illustrated by one participant: *“I started scrolling and saw so many things… But still, couldn’t stop myself.”* (Survivor F). While consultations remained pivotal moments, preferences for communication varied. Some survivors appreciated remote or “only-if-bad-news” communication for its efficiency, whereas others experienced such approaches as impersonal or unsettling.

Intervention opportunities following result disclosure primarily targeted interpersonal and administrative qualities. Enhancing existing touchpoints focused on providing more thorough explanations of scan results and offering opportunities for ad hoc consultation. Augmenting the journey involved introducing additional follow-up options, such as virtual result consultations. Elimination strategies sought to reduce exposure to potentially distressing information by delaying access to scan results in electronic health records until consultation with a healthcare professional. Given the diversity of preferences expressed by participants, flexibility in result communication was considered essential.

### 3.5. Cross-Phase Interventions

Several intervention opportunities were identified as relevant throughout the surveillance journey. Enhancing existing touchpoints primarily targeted interpersonal and administrative qualities through clearer communication, improved use of digital and eHealth platforms, and stronger coordination between healthcare providers. Augmenting the journey focused on introducing additional informational, emotional, and practical support, such as family information sessions, care coordinators, anxiety screening, and psychosocial resources. Together, these intervention opportunities highlighted the need for continuous support across the surveillance journey rather than support limited to individual touchpoints.

### 3.6. Divergent Preferences Across the Cancer Surveillance Journey

While participants shared many experiences of scan-related distress, preferences for support were not uniform. Reminders, digital access to results, and communication formats were experienced as helpful by some participants but distressing by others. During the workshops, participants discussed their differing preferences regarding the communication of scan results. One participant explained: *“I always thought, they could better give me the results by telephone, then I just know and I’m at home. The follow-up consultation could be the next day for the rest of the information.”* (Survivor J). In contrast, another participant noted: *“I find it double. I got the telephone call at my work, so I had to drive home with bad news. I’d rather have them let me come to tell me in person.”* (Survivor G). Similar differences were observed regarding reminders and access to results through digital portals. As Survivor Q stated: “*For blood results, it [digital access] was good, but for example, for my scan, I saw a spot online and I was so distressed. And it was four days until the doctor could tell me it was just due to a cold.*” Although participants differed in their preferences for receiving scan results, digital tools were also viewed as valuable for providing practical information and communicating delays while preserving opportunities for personal interaction when discussing clinically significant results. *“Results should be discussed in person so you can ask more questions. Information and delay communication could be digital, in these times, that should be possible. I fully understand.”* (Survivor I). These contrasting perspectives illustrate how the same touchpoint may be experienced differently depending on individual circumstances and preferences, particularly regarding communication and information delivery. While some valued rapid access to information and the reassurance of early results, others preferred information to be delivered in person where additional explanation and support were available. The findings therefore suggest that patient-centred surveillance care should prioritise flexibility and choice rather than a single standardised pathway, allowing survivors to select communication approaches that best match their needs and preferences.

## 4. Discussion

This study extends the current understanding of scanxiety by demonstrating that scan-related distress is not confined to the imaging procedure itself, but unfolds throughout the broader cancer surveillance journey. By mapping three overarching phases and seven subphases, our findings show that distress emerges through the interaction of emotional, social, and organisational factors throughout surveillance care. Importantly, participants identified sources of distress that extended beyond fear of recurrence or progression, including uncertainty, waiting, fragmented care processes and other challenges related to administrative aspects of care. In response to these challenges, participants identified opportunities to redesign the surveillance journey through improving existing touchpoints, introducing additional sources of support, and removing unnecessary sources of burden. The intervention opportunities addressed interpersonal, technical, environmental, and administrative aspects of care, further highlighting the multidimensional nature of scanxiety and its management. Taken together, these findings suggest that scanxiety should be understood as a journey-wide experience shaped by both individual and system-level factors. Consequently, opportunities to address scan-related distress may be found not only in psychosocial support surrounding imaging, but also in how surveillance journeys are organised, communicated, and experienced over time. Our findings highlight the importance of adopting a broader supportive cancer care perspective that addresses not only emotional, cognitive and behavioural sources of distress and anxiety, but also interpersonal, technical, environmental and administrative sources throughout survivorship [4,7,8,12,13,14,16].

Our findings extend prior scoping and systematic reviews that have predominantly framed scanxiety around the imaging event and emphasised onco-psychological techniques at or near the time of the scan [4,7]. By explicitly mapping subphases (e.g., build-up, logistics, admission, waiting, scan procedure, result waiting, and post-consultation trajectories), we provide a more detailed understanding of how scan-related distress unfolds beyond broad distinctions between before, during, and after the scan, particularly around uncertainty, delays, and result communication. This broader temporal framing resonates with emerging survivorship literature documenting surveillance-associated anxiety throughout follow-up, not merely during imaging encounters [14]. Moreover, it aligns with service research, which has long recognised that experiences unfold across journeys comprising multiple phases, with each phase characterised by its own critical touchpoints and emotional dynamics, and should be studied as such [30,32]. From this perspective, scanxiety can be understood not as a response to a single imaging event, but as a dynamic experience that develops throughout the cancer surveillance journey. Consequently, opportunities to address scanxiety may exist across multiple phases of care rather than at the imaging encounter alone.

Existing scanxiety interventions have predominantly focused on adding psychosocial, educational, or behavioural support around the imaging encounter. Our findings suggest a broader approach to intervention design. Participants identified opportunities to address scan-related distress through three distinct forms of journey redesign: enhancing existing touchpoints, augmenting the surveillance journey with additional touchpoints, processes, or services, and eliminating touchpoints perceived as unnecessary or distress-inducing.

Intervention opportunities aimed at enhancing existing touchpoints primarily focused on improving administrative, interpersonal, and environmental aspects of care. Participants frequently described how fragmented scheduling, unclear admission procedures, delayed result communication, and unsupportive physical environments contributed to uncertainty and distress throughout the surveillance journey. Opportunities such as improving care coordination, clarifying expectations, strengthening communication, reducing noise, and improving wayfinding were viewed as potentially helpful ways of making the surveillance experience more predictable and easier to navigate. These findings are consistent with previous research identifying uncertainty, waiting times, procedural ambiguity, and environmental stressors as important contributors to scan-related anxiety and fear of cancer recurrence [16,50,51,52,53,54,55], while suggesting options for improvement.

Participants also proposed augmenting the surveillance journey through the addition of supportive touchpoints. These included peer support, mindfulness and relaxation strategies, support for companions, and survivor-controlled coping resources. VR-augmented intervention opportunities such as VR-guided relaxation and VR-walkthrough videos were also proposed, illustrating how augmenting the journey could involve introducing new digitally enabled touchpoints. Rather than altering the organisation of care itself, these interventions were intended to provide additional reassurance, emotional support, and coping resources during periods of heightened uncertainty. This aligns with psycho-oncology literature demonstrating the potential value of psychosocial, peer, and practical support in helping individuals manage the emotional challenges associated with cancer surveillance [6,7,8,9,38,56,57,58].

Importantly, participants not only advocated for adding or improving touchpoints. They also highlighted how certain interactions and procedures could themselves become sources of distress. Repeated reminders, fragmented administrative processes, multiple admission steps, and premature access to scan results through digital systems were described as contributing to anticipatory anxiety and cognitive burden. As such, participants identified the removal or simplification of selected touchpoints as a potential strategy for improving the surveillance experience. This finding extends prevailing approaches to scanxiety management by suggesting that distress is shaped not only by individual encounters, but also by complexity and fragmentation embedded within surveillance pathways. By conceptualising elimination as a journey redesign strategy, our study suggests that reducing unnecessary complexity may, in some cases, be as important as introducing additional support. However, eliminating touchpoints may also have unintended consequences, such as increased uncertainty or reduced access to health information, as interactions perceived as burdensome by some survivors may serve as valued sources of information, reassurance, or support for others. Designing patient-centred surveillance journeys may therefore involve not only creating new touchpoints, but also critically evaluating which existing touchpoints genuinely add value for survivors and companions.

An important finding of this study is that administrative quality may play a key role in shaping experiences of scan-related distress. Within Dagger et al.’s framework [48], administrative quality encompasses the subdimensions of timeliness, operations, and support. Although existing scanxiety research has predominantly focused on psychosocial and supportive interventions, participants also frequently described intervention opportunities related to the timeliness and operational aspects of surveillance care, including appointment scheduling, coordination of work and family responsibilities, admission procedures, hospital navigation, waiting times, and result communication. While scanxiety is often conceptualised as arising from fear of recurrence, uncertainty, and the imaging procedure itself, our findings suggest that the administrative aspects of care may also affect emotional well-being throughout surveillance [59]. Uncertainty, practical burden, and fragmented processes were linked to heightened distress, and participants identified multiple opportunities for improvement in these areas. These findings suggest that healthcare processes may function as supportive care resources when they help reduce uncertainty, burden, and distress. Importantly, these administrative challenges were described across multiple phases of the cancer surveillance journey, from scheduling and preparation to admission procedures and result communication, indicating that opportunities for administrative improvement may exist throughout the surveillance pathway rather than at a single point of care. Taken together, these findings complement the predominantly psychosocial and informational focus of existing scanxiety interventions and suggest that administrative improvements represent promising targets for future intervention development and evaluation [7,17,18,19,20,21,22,23,24,25,26,27,28].

Although only a small number of companions participated in this study, their accounts suggest that scanxiety affects not only cancer survivors, but may extend to their companions. They described experiencing distress throughout surveillance care, particularly during periods of uncertainty and while waiting for scan results, supporting emerging evidence that scanxiety has a relational dimension [33]. Interestingly, some companions appeared hesitant to characterise these experiences as scanxiety because they did not undergo the scan themselves and perceived survivors as carrying the primary burden of illness. This suggests that the impact of surveillance care may extend beyond survivors and that distress experienced by companions may not always be fully recognised or reported. Despite their limited number, their inclusion in the co-creation process contributed perspectives that may otherwise have remained overlooked. Moreover, explicitly incorporating companions into the research design encouraged survivors and healthcare professionals to reflect on the experiences, needs, and support requirements of companions throughout the surveillance journey.

Finally, participants differed considerably in their preferences throughout the surveillance journey. Touchpoints that were perceived as reassuring by some participants were experienced as distressing by others. For example, reminders, access to digital results, and communication formats were valued differently among survivors. This suggests that the effectiveness of interventions may depend not only on the intervention itself, but also on the context and preferences of the individual receiving it. Several participants also identified opportunities for interventions that could be facilitated through digital health technologies, such as patient portals, automated communication systems, and digital, tailored information resources. However, not all participants saw digital approaches as desirable, highlighting variation in digital preferences and supporting previous research suggesting that digital solutions should complement rather than replace face-to-face support. Preference differences may reflect variation in information needs, confidence in using digital technologies, the value individuals place on direct interaction with HCPs, as well as the individuals’ approaches to managing uncertainty [60,61,62,63,64]. Although these factors were not explicitly explored in the present study, they may help explain why survivors differed in their preferences for receiving information and support throughout the surveillance journey. Consequently, rather than pursuing a single optimal surveillance pathway, healthcare organisations may benefit from offering flexible communication and support options. By allowing survivors to engage with surveillance in ways that align with their individual needs, preferences, and circumstances, healthcare organisations may facilitate a more person-centred surveillance experience and potentially improve survivors’ overall experience of care.

### 4.1. Practical Implications

The findings of this study suggest that efforts to reduce scanxiety should extend beyond psychosocial support delivered around the time of the scan. Participants identified important opportunities for improvement across interpersonal, environmental, administrative, and technical qualities of care. As such, healthcare organisations should adopt a broader supportive care approach to scanxiety management that combines emotional and psychosocial support with improvements in communication, care coordination, appointment scheduling, waiting times, result delivery, and appropriately tailored digital tools. Importantly, supportive care efforts should consider both survivors and companions, recognising that surveillance-related distress may affect both members of the survivor-companion dyad and that companions may also have support needs throughout the surveillance journey.

The co-created intervention opportunities identified in this study may serve as potential strategies for redesigning surveillance care through enhancing existing touchpoints, augmenting the journey with additional touchpoints and services, and eliminating unnecessary or distress-inducing touchpoints. Particular attention should be paid to phases of the cancer surveillance journey characterised by uncertainty, waiting, or fragmented care processes, as these emerged as important sources of distress. However, these opportunities require future evaluation to determine their feasibility, acceptability, and potential impact.

The findings also demonstrate the value of co-design in identifying patient-centred opportunities for improving surveillance care. Involving survivors, companions, and healthcare professionals together enabled the development of intervention opportunities that reflected both lived experiences and care delivery realities.

### 4.2. Strengths and Limitations

A key strength of this study lies in its participatory design, which actively involved cancer survivors, their companions, and healthcare professionals in co-designing intervention opportunities. This approach ensured that the findings were grounded in lived experience and contextually relevant across diverse phases of the cancer surveillance journey. The inclusion of multiple cancer types and stakeholder perspectives enhances the transferability of the results to broader survivorship contexts. Additionally, the use of service design methods such as journey mapping and the 6-3-5 brainstorming technique allowed for a rich exploration of emotional and procedural pain points. A further strength is the operational mapping of the cancer surveillance journey into phases and subphases, paired with co-created opportunities for journey redesign through enhancement, augmentation, and elimination of touchpoints.

However, several limitations should be acknowledged. The study was conducted within a single geographic region (Flanders, Belgium), which may limit generalisability to other health service systems or cultural contexts. While the qualitative design provided depth, it does not allow for statistical inference or measurement of intervention effectiveness. The co-creation workshops relied on self-reported experiences, which may be subject to recall bias or social desirability bias. Recruitment was partly convenience-based and may have underrepresented certain groups, including individuals with lower health literacy and linguistic minorities. In addition, the small number of participating companions and absence from the second round focus group limit the extent to which the identified experiences and intervention opportunities reflect companion data saturation and the diversity of companion perspectives. Finally, the proposed intervention opportunities have not yet been implemented or evaluated in clinical practice, and future research is needed to assess their feasibility, acceptability, and impact on scanxiety reduction.

## 5. Future Research

Future research should focus on evaluating the effectiveness, feasibility, and implementation of the co-created interventions identified in this study in the context of routine survivorship care. In particular, studies are needed to examine their impact on patient-centred outcomes, such as scanxiety, psychosocial well-being, health-related quality of life, experiences of care, and companion outcomes.

Additionally, future studies should investigate how scanxiety and support preferences vary across surveillance contexts, cancer types, and patient groups. Given the variation in preferences identified in this study, future work should explore how surveillance services can be designed to provide greater flexibility and choice while remaining feasible to implement in clinical practice. This may be particularly relevant for digital health solutions, as participants expressed differing preferences regarding digital reminders, access to results, and communication channels. Future research should therefore investigate how digital support can be tailored to individual information needs, coping styles, and preferences for engagement with digital support. In addition, the experiences and support needs of companions warrant further investigation, as they remain relatively underexplored within the scanxiety literature.

Future research should examine how healthcare organisations can reduce scanxiety through the design of surveillance services. Our findings suggest that distress is shaped not only by psychosocial factors but also by organisational processes such as coordination, scheduling, communication, and waiting times, highlighting opportunities to embed supportive care within routine follow-up.

Finally, longitudinal research could improve understanding of how scanxiety evolves across repeated surveillance cycles and over the survivorship trajectory. Such work may help clarify how previous scan experiences influence future expectations, coping strategies, and preferences for support, thereby informing the development of more responsive and patient-centred surveillance pathways.

## 6. Conclusions

Scanxiety is a recurring experience that extends beyond the imaging procedure and unfolds throughout the cancer surveillance journey. By mapping this journey and co-designing intervention opportunities with survivors, companions, and healthcare professionals, this study highlights how scan-related distress is shaped by interpersonal, environmental, technical, and administrative aspects of care.

The findings suggest that scanxiety should be understood as a supportive care challenge that affects cancer survivors throughout the cancer surveillance journey. Participants identified opportunities to redesign surveillance care by enhancing existing touchpoints, augmenting the journey with additional touchpoints and services, and eliminating unnecessary or distress-inducing touchpoints. The variation in preferences observed across participants further suggests that patient-centred surveillance care requires flexibility rather than a single standardised approach.

By adopting a journey perspective, this study provides a foundation for the future development and evaluation of supportive care interventions that address the interpersonal, environmental, technical, and administrative factors that shape scanxiety throughout survivorship.

## Figures and Tables

**Table 1:** Overview of the workshops.

	Participant Group	Content of the Workshop		Number of Sessions	Total Number of Participants
Co-creation workshop round one	Cancer survivors & their companions	Part one: Journey mapping	Part two: 6-3-5 brainstorm exercise	*n* = 6	*n* = 20
	HCPs	Part one: Immersive exercise using patient journey map		*n* = 3	*n* = 27
Co-creation workshop round two	Cancer survivors, their companions & HCPs	Part one: Ranking questionnaire of interventions and impact of scanxiety	Part two: Focus group discussing results	*n* = 1	*n* = 31 participants from round one plus five additional eligible candidates filled in the questionnaire.

**Table 2:** Redesign approaches with intervention opportunity examples and the redesign approach coding.

Redesign Approach	Definition	Illustrative Intervention Opportunity Examples	Redesign Approach Coding	Quality Dimensions *			
				(I)	(T)	(E)	(A)
**Enhancement**	Improving an existing touchpoint, process, interaction, environment, or resource already present in the cancer surveillance journey.	Providing better guidance during preparation (Workshop D)	Enhancing existing preparation touchpoints by providing better guidance to patients	X			X
		Communication during the examination (Workshop C)	Enhancing existing care interactions by improving communication with patients during the examination	X			
		Better use of digital platforms (Workshop D, H)	Enhancing the existing digital environment by improving information and communication with patients	X		X	
		Privacy in the waiting room and comfortable waiting facilities (Workshop C)	Enhancing an existing physical environment by accommodating patients through greater privacy and comfort in waiting areas			X	
		No trivial conversations with other patients about “minor” conditions (Workshop B)	Enhancing the existing waiting-room environment by accommodating patients through reducing distressing social interactions	X		X	
		Staff education on scanxiety (Workshop G)	Enhancing existing professional care interactions by improving professionals’ awareness of scanxiety to better support patients	X	X		
**Augmentation**	Adding a new touchpoint, support mechanism, resource, or service that was not previously part of the surveillance journey.	Telephone contact the day before the examination (Workshop A, G)	Adding a new supportive touchpoint by introducing telephone contact before the examination	X			X
		Assessing anxiety beforehand through questionnaires (Workshop D, G)	Adding a new screening touchpoint by measuring anxiety beforehand through questionnaires		X		X
		The patient should be able to contact a care coordinator, nurse specialist or case manager (Workshop I)	Adding a new support service and communication channel by enabling contact with a care coordinator, nurse specialist, or case manager	X			X
		A buddy system throughout the trajectory (Workshop D)	Adding a new peer-support mechanism by introducing a buddy system throughout the surveillance journey	X			X
		Testimonials on the website from peer experts (Workshop G)	Adding a new experiential information resource by providing patient testimonials from peer experts			X	X
		Information sessions for the whole family (Workshop G)	Adding a new educational and support service by providing information sessions for the whole family	X			X
		Free yoga or mindfulness sessions (Workshop D, G)	Adding a new coping-support resource by providing free yoga or mindfulness sessions				X
**Elimination**	Removing touchpoints perceived as unnecessary, burdensome, redundant, or distress-inducing.	Making the registration process less fragmented (Workshop C)	Eliminating unnecessary administrative touchpoints by reducing fragmentation in the registration process				X
		Release results in EHR only after consultation (Workshop C, D, F, G)	Eliminating exposure to a distress-inducing information touchpoint by releasing results only after consultation				X
		One reminder is sufficient (Workshop A, E)	Eliminating redundant reminder touchpoints by limiting communication to a single reminder				X
		Examinations and discussion of results on the same day (Workshop B)	Eliminating additional visits, logistical burden and prolonged waiting periods by scheduling examinations and result discussions on the same day				X
		Reducing waiting time for results (All workshops with patients, companions and healthcare professionals)	Eliminating part of the waiting period by reducing the time between examination and receipt of results				X
* Quality dimensions	**(I) Interpersonal quality**: Quality of interactions between patients, companions, and healthcare professionals, including communication, empathy, emotional support, and trust.						
	**(T) Technical quality**: Quality of professional expertise, clinical competence, and technical execution of surveillance care.						
	**(E) Environmental quality**: Quality of the physical and digital tangibles and the environment in which cancer surveillance care is experienced.						
	**(A) Administrative quality**: Quality of organisational and process-related aspects of care, including information provision, coordination, scheduling, accessibility, non-clinical and emotional support and timeliness.						

## Data Availability

The datasets generated and/or analysed during the current study are not publicly available due to privacy and ethical considerations but are available from the corresponding author upon reasonable request.

## Supplementary Materials

The following supporting information can be downloaded at https://www.mdpi.com/article/10.3390/healthcare14203536/s1: Table S1: Participant Demographics; Table S2: Extended table of intervention opportunities; File S1: Ranking Questionnaire.
