# Supplementary material for: Addressing Scanxiety Throughout the Cancer Surveillance Journey: A Co-Design Study
Source: Healthcare (Basel). 2026 Oct 20;14(20):3536. doi: 10.3390/healthcare14203536 (PMC13704750; doi:10.3390/healthcare14203536)
Supplement: Supplementary file 1 [file healthcare-14-03536-s001.zip › File S1 Ranking Questionnaire.pdf]

Scanxiety Ranking Questionnaire

Dear participant,
Thank you for your interest in the research on scanxiety. In the first phase, we collected a large number of ideas about possible interventions that could help reduce scanxiety at different moments. We have now processed the results from this first phase and are moving on to a second phase.
In this second phase, we aim to further develop the interventions that have the greatest potential impact for patients and their loved ones. To help us determine what to focus on, we kindly ask you to complete this questionnaire. Completing the questionnaire will take a maximum of 10 minutes.

The data are collected within the framework of the Scanxiety project, approved by the Ethics Committee of Ghent University Hospital (reference number: ONZ-2024-0177).
In accordance with the General Data Protection Regulation (GDPR) (EU) 2016/679 of 27 April 2016 and the Belgian Act of 30 July 2018 concerning the protection of natural persons with regard to the processing of personal data and the free movement of such data, your privacy will be respected and you will have access to the data collected about you. Any errors may be corrected upon your request. Your other rights, including the right to restrict the processing of your (personal) data, the right to have your data erased under certain circumstances, and the right to lodge a complaint, are also guaranteed.
For more information about your rights and how to exercise them, please visit:
https://www.ugent.be/nl/univgent/privacy/privacyverklaring.htm
The Data Protection Officer can also provide further information regarding the protection of your personal data if required.
Contact details: Hanne Elsen privacy@ugent.be
☐ I consent to participate in this study.

Please enter your name here

____

I am:
○ Patient
○ Relative / Family Member / Friend
○ Healthcare Professional

Show this question
If I am: = Patient

What is your diagnosis?

____

Show this question
If I am: = Healthcare professional

What is your professional role?

____

Show this question
If I am: = Relative

What is the diagnosis of your partner/family member/friend?

____

I previously participated in a scanxiety workshop during Phase 1?

◯ No

◯ Yes

Show this question

If I am: = Patient

Please indicate, using the slider, which moments you experience as the most burdensome in relation to scanxiety.

.

	From recieving the appointment reminder until the day of the scan	Transfer to the hospital	Admission and waiting in the waiting room	Undergoing the scan itself	Waiting at home for the results	Consultation with the physician
Most burdensome moment (1)						
Second most burdensome moment (2)						
Third most burdensome moment (3)						

Show this question

If I am: = Healthcare professional

Please indicate, using the slider, which moments you believe are experienced as most burdensome by patients and/or their relatives in relation to scanxiety.

	From recieving the appointment reminder until the day of the scan	Transfer to the hospital	Admission and waiting in the waiting room	Undergoing the scan itself	Waiting at home for the results	Consultation with the physician

Most burdensome moment (1)	
Second most burdensome moment (2)	
Third most burdensome moment (3)	

Show this question

If I am: = Relative

Please indicate, using the slider, which moments you experience as most burdensome in relation to scanxiety.

	From recieving the appointment reminder until the day of the scan	Transfer to the hospital	Admission and waiting in the waiting room	Undergoing the scan itself	Waiting at home for the results	Consultation with the physician
Most burdensome moment (1)						
Second most burdensome moment (2)						
Third most burdensome moment (3)						

Please, briefly explain your answer below

We have grouped the intervention ideas into different categories. These categories have been organised according to the period in which they may have an impact: (1) the period before the scan day, (2) the scan day itself, (3) the period after the scan, and (4) overarching interventions. To help us select interventions for the next workshop, we ask you to rank these categories in order of importance for improving the current scan pathway and reducing scanxiety. Examples are provided in brackets.

Rank the following categories of interventions aimed at the period before the scan day

_____ Planning tailored to the patient (self-scheduling, more flexible scheduling, efficient scheduling)
_____ Improved information provision beforehand (becoming familiar with the procedure through VR or a video, knowing what the care pathway will look like)
_____ Personalised preparation procedure (a single reminder message, contact beforehand regarding psychological or practical matters)
_____ Accessibility (improving physical access to the hospital and telephone or digital accessibility for questions during preparation)

Rank the following categories of interventions aimed at the scan day

_____ Waiting room (environment, activities/distractions)
_____ Reducing waiting times (before the scan and during preparation)
_____ Improving communication and interaction by staff (possibility to communicate during MRI, clear communication about what is happening during the scan)
_____ Improving physical comfort during the scan (less noise, greater comfort while lying down, open scanner)
_____ Providing distraction during the scan (VR, video, music)
_____ Support (staff, volunteer, or caregiver)
_____ Providing a small token after the scan (cafeteria voucher, small care product)
_____ Providing a preliminary result immediately after the scan (e.g., when an ultrasound is performed by a radiologist)
_____ Transparency about waiting times (verbal updates, displaying waiting times on a screen in the waiting room or via the hospital app)
_____ Improving preparation for the scan or examination (providing a bathrobe instead of a hospital gown, calming music in the PET scan waiting area, not requiring patients to undress too early)
_____ Pharmacological support (anxiety-reducing medication for patients with severe anxiety before entering the scanner, sedation for PET-CT)
_____ Supportive services (childcare during the scan or consultation)
_____ Parking (free parking, parking close to the radiology department)

Rank the following categories of interventions aimed at the period after the scan

_____ Optimising the use of eHealth platforms (guidance for patients, delaying release of images until the report or consultation is available, etc.)
_____ Tailoring the way results are communicated to patient preferences (telephone consultation, face-to-face consultation, etc.)
_____ Minimising the time between the scan and receiving results (additional multidisciplinary meetings, same-day scans and results)
_____ Improved follow-up after the scan (consultations, proactive telephone contact, etc.)

Rank the following categories of interventions across the scan journey

_____ Learning to cope with scanxiety (coping strategies, relaxation techniques)
_____ Increasing involvement of peer support networks (buddy systems, support groups)
_____ Improving transparency throughout the pathway (pathway steps, timelines)
_____ Professional support throughout the entire pathway (oncology coach, general practitioner, psychologist and/or nurse specialist, multidisciplinary counselling, transdisciplinary collaboration)
_____ Involving and supporting family members and loved ones (information sessions for the whole family, the opportunity to bring a trusted person to appointments)
_____ Raising awareness of scanxiety among patients and healthcare professionals (screening for scanxiety, training for healthcare professionals, patient diaries)
_____ Practical support throughout the pathway (sick leave arrangements, parking, financial support)

Would you like to participate in an online workshop in which we will further develop these interventions?
○ Yes
○ No
